# Supplementary material for: The Oncological Implication of Sentinel Lymph Node in Early Cervical Cancer: A Meta-Analysis of Oncological Outcomes and Type of Recurrences
Source: Medicina (Kaunas). 2022 Oct 27;58(11):1539. doi: 10.3390/medicina58111539 (PMC9698841; doi:10.3390/medicina58111539)
Supplement: Supplementary file 1 [file medicina-58-01539-s001.zip › medicina-1934152-supplementary.pdf]

## Supplementaries

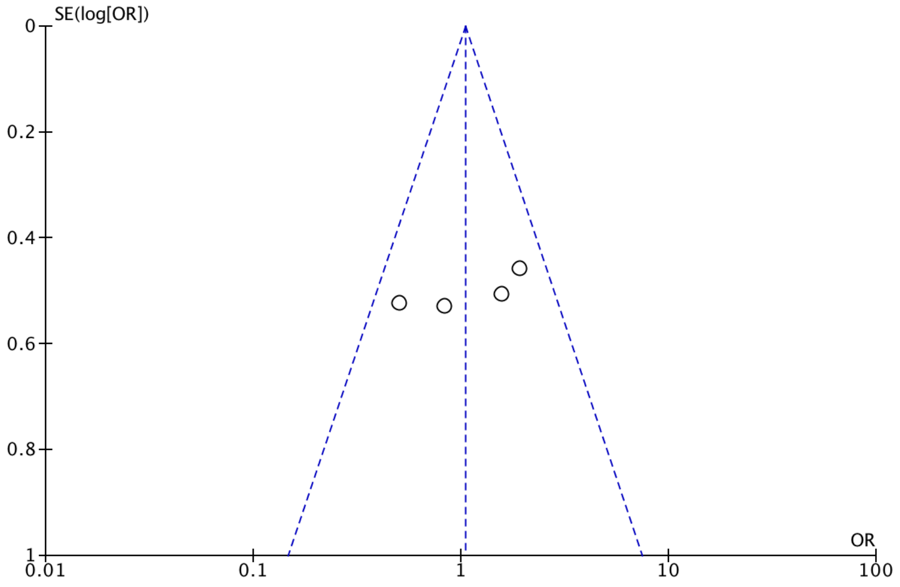


**Figure S1.** Funnel Plot DFS.

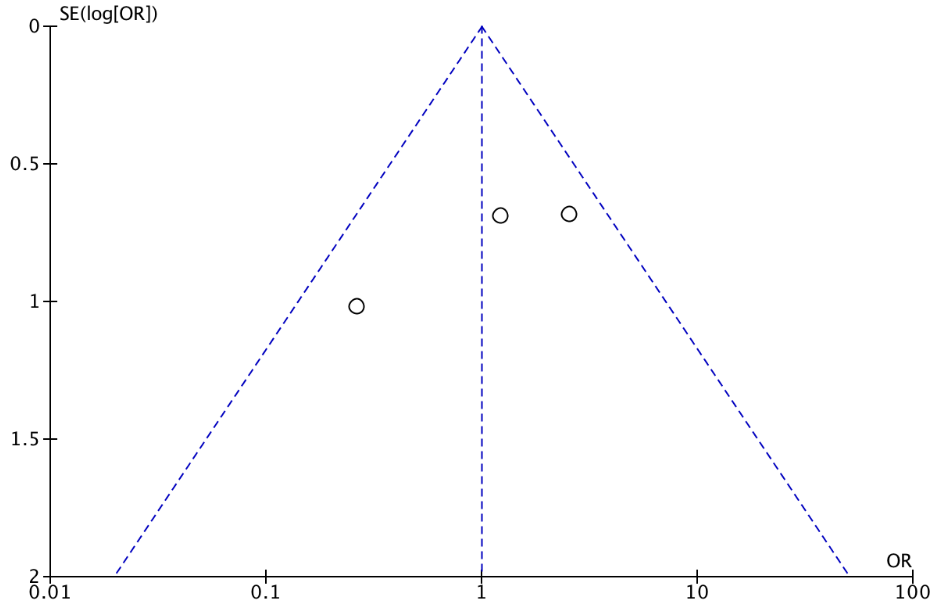


**Figure S2.** Funnel Plot OS.

**Table S1.** Newcastle–Ottawa scale.

| Comparative Studies | | | | | | |
|---|---|---|---|---|---|---|
| **Name** | **Country** | **Study design** | **Selection** | **Comparability** | **Exposure** | **Tot** |
| Balaya2022 [14] | France | Retrospective Case-Control Multicentric study | 1 | 1 | 2 | 4 |
| Favre2021 [15] | France | Prospective Randomized Multycentric study | 2 | 2 | 2 | 6 |
| Gortzak-Uzan2010 [16] | Canada | Retrospective Case-Control Monocentric study | 2 | 2 | 2 | 6 |
| Lennox2016 [17] | Canada | Retrospective Case-Control Monocentric study | 1 | 2 | 2 | 5 |
